# Deciphering equity style returns: An analysis of size and value anomalies in the Pakistani stock exchange

**DOI:** 10.1016/j.heliyon.2023.e19022

**Published:** 2023-08-08

**Authors:** Muhammad Kashif, Sumaira Chamadia, Farhan Ahmed, Juan E. Trinidad Segovia

**Affiliations:** a Faculty of Management Sciences, SZABIST University, Pakistan; b Economics & Management Sciences Department, NED University of Engineering and Technology, Karachi, Sindh, Pakistan; c Department of Economics and Business, Universidad de Almeria, Spain

**Keywords:** Style investing, Size anomaly, Value anomaly, Business cycles, Fama-French three factors model

## Abstract

This study aims to identify the underlying causes of variation in the time series and cross-sectional equity style returns in the emerging stock market of Pakistan. We use asset pricing models and incorporate variables reflecting business cycle fluctuations to assess the time-varying size and value premiums. The methodology of this paper involves constructing style portfolios based on firm-specific characteristics such as market capitalization, price to earnings ratio, book-to-market equity ratio, momentum, and asset growth. We find that the style portfolios earn abnormal returns consistently which cannot be explained either through asset pricing models or business cycles variables. However, the size and value premiums are found to be subsided during the troughs of economic cycles. The results suggest that the abnormal returns for style portfolios are likely driven by firm-specific characteristics rather than macroeconomic factors. Overall, this study contributes to the literature on style investing by providing insights into the profitability of equity style portfolios in the Pakistani equity market. Our findings have implications for stock picking, investment management and risk factor analysis.

## 1 Introduction

The efficient market hypothesis posits that all publicly available information is instantaneously and accurately incorporated into the stock prices and thus there is no unadjusted information content in the security prices. However, equity styles based on certain firm specific characters such as small market capitalization, low price to earnings or high book to market equity ratio, momentum and asset growth have been empirically evidenced to outperform the market by several researchers including [[1], [2], [3], [4], [5]] and a few others. Style investing is based on the premise that instead of selecting specific stocks for investment, a specific style is chosen to construct portfolios.

Although equity style investing has been widely recognized as a profitable strategy for portfolio investment in the literature, the underlying cause of such arbitrage is debatable in academics. The phenomenon of style investing, which reports that a group of assets is able to better earn than other sets of stocks under the same economic conditions, is abstruse and the academia has not been able to find unanimously the fundamental theory underlying this phenomenon. The identification of financial anomalies is seen as an antithesis to the semi-strong form of efficient market hypothesis (EMH) by the scholars and thus has fomented a rigorous deliberation on the credibility and the plausible reasons of style premiums. Further, the style premiums have shown to be reversed in the recent research studies as compared to the original ones. These discrepancies have led the scholars to hypothesize that the style premiums tend to follow a pattern that can either be explained by the fundamental characteristics or some exogenous factors. Some researchers argue that stock returns should have a positive correlation with the business cycles [6,7] because of the impact of business peaks and troughs on the cash flows of the firm and the interest rates. It represents the causal link between the expectations of future economic conditions with the future stock returns. Another school of thought suggests that the return differential for size and value is a result of individual firm-specific characteristics of the stocks rather than the style differential [8].

Although there are a number of studies conducted to investigate the impact of business conditions on the size and value premium in developed and developing stock markets, little research has been done in Pakistani stock exchange to assess the size and value anomaly and its persistence in varying business conditions. Thus, we attempt to contribute to the literature by identifying the underlying causes of the variation in the time series and cross-sectional equity style returns. We conduct an extensive study on style returns in the Pakistan Stock exchange by applying the asset pricing models as well as incorporating the variables reflecting the business cycle fluctuations to assess the time varying size and value premiums. We report that the style portfolios earn abnormal returns consistently which is not explained through either Fama-French three and five factors models or the business cycles model.

In the first section, detailed introduction has been discussed. The rest of paper comprises of literature review in second section, methodology; third section, empirical findings and discussion in fourth section and conclusion; fifth section.

## 2 Literature review

Equity styles can be defined as the grouping of stocks based on similar quantifiable characteristics. For example, size of the firm, growth rate, value and the firm's beta. The mentioned characteristics are widely used in the investment community in constructing portfolios however the list is not exhaustive. Investors also follow feedback trading strategies that take momentum into account while constructing portfolios that is taking a long position on past winners and short on past losers. The extant anomalies in the financial markets suggest that investors can beat the market by systematically following and rotating various equity styles in asset allocation. The emergence of style investing dates back to the 1970s when some researchers (For example, King [9] and Farrell [10] found that stocks and portfolios that possess identical characteristics exhibit similar return patterns [11]. The equity style and style investment have gained significant attention among investment practitioners due to several reasons. First, evidence from the literature shows the pattern of portfolio returns is determined largely by the investment style it possesses. For example, a study by Brinson, Singer and Beebower [12] reports that 90% of the variation in the returns is attributed to the asset allocation strategy adopted by the pension funds. Similarly, Hansen [13] found 60% of the return differential in the short to medium term due to the investment styles. In the style analysis conducted by Sharpe [14] it was claimed that around 90% of the monthly variation in returns was due to the style investing and the source of remaining 10% was the idiosyncratic attributes of the equities held in the portfolio that is the selection of specific stocks by the fund managers. Similarly, the capital asset pricing model by Lintner [15] and Sharpe [16] has been a widely recognized theory in asset pricing, the empirical studies found that incorporating market risk alone in the model cannot fully explain the stock returns. The academic studies in the highly diversified markets of the USA, Europe and other developed economies found negative premiums on market beta despite the positive returns on the market portfolio and thus engendered an argument that the stock return premiums cannot be fully explained by adjusting the systematic risk only [17]. The two propositions given by CAPM that the intercept (α) is zero and beta is the only factor explaining the excess returns has been questioned by the scholars because of the model's failure to capture the variation in the cross-sectional returns.

The investor community has therefore attempted to identify the factors which may serve against non-diversifiable risk in order to explain the variation in expected return premiums. The pioneering studies in this regard are accredited to Basu [18] who used price to earnings multiple to investigate the excess returns and Banz [19] who used the market capitalization to capture the impact of size effect on the return premiums in addition to the market beta in the US equity market. Acharya and Pedersen [20] and Amihud [21] identified liquidity risk as compensation for holding small stocks whereas Carlson, Fisher and Giammarino [22] and Garleanu, Panageas and Yu [23] attributed the risky growth options as the reason for size premium. More recently Asness, Frazzini, Israel, Moskowitz and Pedersen [24] reported a significant size effect after controlling for the firm's quality measured through profitability, growth, stability and safety. On the other hand, value premium emerges as a result of investors' perception of strong fundamentals which are incorrectly undervalued in the market now and will generate abnormal returns when the prices increase whereas growth stocks are perceived to be overvalued because of their growth potential [25]. In this regard, Clark and Qiao [26] attribute value premium as a compensation for bearing financial inflexibility which can be defined as the firm's inability to alter or adjust the investments to cope up with the exogenous shocks. They report that the value firms are more financially inflexible than growth firms and hence earn an excess return.

There are some other studies found in the literature which reported the impact of the book to market ratio [27], cash flow to price and historical trend of sales growth on the stock returns [4,28]. Fama and French [3] and Fama and French [29] have presented the most eminent work in this regard by using a multifactor model widely recognized as three factors model. Although the size and book to market might not be theoretically justified to assume the unidentified risks, the inclusion of these factors significantly improved the explanatory power of the model to explain the cross-sectional variation in expected returns.

Equity style investing has been favored equally by fundamental investors and the technical analysts however the regularity of return by these styles is disturbed in certain economic periods. It has been noted empirically that the small size stocks and value stocks show cyclical patterns different from the overall market. Evidence in the US market suggests that the size premium has ceased to exist since the 1980s [3,30,31]. Several other studies found the absence of size effect in the UK market after the 1980s [32,33] and recently in the emerging markets [34]. Fama and French [35] in their recent study have also confirmed the absence of size premium in the four markets including Japan, Europe, North America, and the Asia Pacific since 1990. Horowitz, Loughran and Savin [36] have also verified the cyclicality of size premium suggesting that the impact of size premiums is strong in one period and it weakens or even disappears in the other period. Similarly, the value premiums are more pronounced in the longer investment horizons as compared to the shorter holding periods [37]. In this regard, Ahn, Min, and Yoon [38] report that although the unconditional size effect has disappeared after 1980s, the conditional premium depending on the economic cycles is still present. Several other studies have also found an association between size and value-group premiums and the economic cycles in the US [37,39] and UK markets [40]. The findings of Hou and van Dijk [41] also confirmed that the disappearance of size premium is due to the unexpected shocks to the profitability of small and large firms. They premise that the negative cash flow shocks depress the returns on small firms whereas the positive cash flow shocks contributed to the higher than expected returns for the large firms. Ahn, Min, and Yoon [38] attributed the unexpected shocks to the various stages of business cycles.

The researchers have given two plausible economic rationales for the time-varying style premiums. The first explanation rests upon the noise created by the speculative traders which leads to asset pricing bubbles, fads, and subsequent market crashes. However, a large body of literature relates the varying style premiums to the changes in macroeconomic variables. The expected returns are likely to react to the structural changes in the economy and the economic cycles [42,43]. Nevertheless, all the stocks are not equally sensitive to the changes in the economic cycles; different equities react differently over the phases of business cycles depending on the nature of their business. For example, the defensive stocks are not much affected by the business cycles’ boom and bust, whereas the firms producing luxurious and capital goods show more sensitivity towards the variation in the business cycles. Similarly, firms with small market capitalization are affected more in recession. On the other hand, value stocks tend to lure more benefits when the economy is in the transition phase from the weakness to the recovery arena whereas growth stocks show the completely reverse pattern that is they outperform the value stocks in a slow-growing economy [44].

The firm's exposure to risk and the ability to produce the expected cash flows generally varies according to the business cycles. Markets face lower risks during the peak and higher risks during the trough and consequently the market risk premium is lower in the economic boom and higher in the recession [45]. Similarly, the yield curve indicates the volatility of the business cycle and the term structure also determines the cost of capital for a firm. Both Chan & Chen [27] and Fama & French [29] support the proposition that distressed stocks are more sensitive to the fluctuation in the economic phases. Further, they suggest that the returns of such stocks are driven by the above mentioned macroeconomic variables in addition to the access to credits. Few studies (For example, Bernanke & Gertler [46], Hahn and Lee [47] and Kiyotaki & Moor [48]) suggest that changes in the conditions of credit markets can have varied effects on the risk and returns of various style portfolios.

According to Campbell [49], the variables indicating the time-varying business opportunities should be able to forecast the market returns as well as able to gauge the patterns of stock returns in the cross-section. There are numerous variables studied earlier which are used to measure the business cycles including the GDP growth rate, industrial production, inflation rate, term structure, default premium, and the yield curve. Changes in the credit market can have different effects of risk exposure of the firms depending upon their size. The asymmetrical information inherent in borrowing for the credit markets results in agency cost which requires firms to use collateral while acquiring funds. Large firms have more collateral than the smaller firms and thus are in a better position to raise funds externally and hence the period of lower liquidity and high cost of borrowing is expected to affect small firms more adversely [50]. It can be inferred from this argument that the small firms reduced net worth in a tighter credit market will lead to the increased agency cost which in turn would decrease the collateral resulting in the reduced access to the credits during recession because the investors would follow “flight to quality” and attracted towards the large firms which are comparatively less risky than the small firms [46]. Fama and French [7] have also confirmed that the effect of business cycles on the stock returns are larger for the small-sized firms. Their study found that the value-weighted portfolio which gives more weight to larger firms is less sensitive to changes in business conditions.

In sum, the existing literature suggests that equity styles, which are based on quantifiable characteristics such as firm size, growth rate, value, and beta, can be used to construct portfolios that outperform the market. Additionally, feedback trading strategies, which take momentum into account, can also be used to beat the market. Various factors such as size, value, and liquidity have been identified as contributing to return premiums. The CAPM model's failure to fully explain stock returns has led researchers to search for other factors that may serve against non-diversifiable risk. The literature has identified several factors such as liquidity risk, growth options, and financial inflexibility that contribute to return premiums. The research gap in this literature is that it is unclear how these equity styles perform in the Pakistani stock market, as there is a lack of research on this topic in the context of Pakistan. Furthermore, it is not known how the various factors that contribute to return premiums in the US market apply to the Pakistani stock market. Therefore, this study aims to fill this gap by examining the performance of various equity styles in the Pakistani stock market and identifying the factors that contribute to return premiums in this market.

## 3 Data and methodology

### 3.1 Data

We take monthly returns of all non-financial firms of the Pakistan stock exchange from the Thomson Reuters data stream. The data for the firm-specific characteristics which consists of market capitalization market-to-book value and the industrial production index used to measure the business cycle patterns are also taken from the Thomson Reuters data stream. The sample size covers a span of 14 years from January 2004 till December 2017. In order to avoid the survivorship bias, we have included all listed as well as delisted companies because the dead companies might have captured the style anomalies while these were operational. The non-financial companies are selected for our sample because the ratios of financial sector companies have different interpretations as compared to non-financial companies (Fama & French [51]). We begin with the 889 companies listed on Pakistan stock exchange which were then screened to include only non-financial firms of 655 firms.

We construct the style portfolios by sorting the fundamental characteristics in ascending order then categorized into quintiles. Quintiles are arranged in a way that quintile 1 has the lowest and quintile 5 has the highest value of a given fundamental characteristic. Further, the number of stocks in each quintile is the same and longing Quintile 1 and shorting quintile 5 will give hedge portfolios. The hedge portfolios are constructed in equally weighted and value-weighted styles.

### 3.2 Constructing alphas for the portfolios

We use the Generalized method of moments (GMM) as the econometric methodology to estimate the Alpha's of the portfolios.

(1) $$R_{i},=\alpha_{i}+\beta_{i}F+{\varepsilon_{i}}_{,t}\left(t=1...T,i=1...N\right)$$

where

R = Excess return on portfolio i in time period t.

N = Number of portfolio.

T = Time period.

F=K * 1 vector of excess return factor portfolio.

B = Vector of beta's.

This equation assumes that the excess returns of the portfolio are linearly related to its beta's. For simplicity sake the above equation is restated as follows:

(2) $$R_{tx}=\alpha_{i}+\beta_{i}\left(f_{t}\right)+\varepsilon \left(t\right)$$

where, E(***ε****t*) = 0 and Cov (*f t*,***ε****t*) = 0 t = 1 … …T.

Where $Rtx=\left[\begin{array}{l} R_{1}\\ .\\ .\\ R_{10} \end{array}\right]$ is a 10 × 1 vector containing excess return of the ten decile portfolios, $\alpha =\left[\begin{array}{l} \alpha_{1}\\ .\\ .\\ \alpha_{10} \end{array}\right]$ is a 10 × 1 vector containing the intercept of the model and $\beta =\left[\begin{array}{l} \beta_{1}\\ .\\ .\\ \beta_{10} \end{array}\right]$ is the 10 × 1 vector which measures the sensitiveness of the portfolios to the market portfolio. F is the excess market return and $\varepsilon \left(t\right)=\left[\begin{array}{l} \varepsilon_{1}\\ .\\ .\\ \varepsilon_{10} \end{array}\right]$ is the error matrix. Thus the equation can be written as follows:

$$Rtx=\left[\begin{array}{l} R_{1}\\ .\\ .\\ R_{10} \end{array}\right]+\left[\begin{array}{l} \alpha_{1}\\ .\\ .\\ \alpha_{10} \end{array}\right]+\left[\begin{array}{l} \beta_{1}\\ .\\ .\\ \beta_{10} \end{array}\right]*\left[F\right]+\left[\begin{array}{l} \varepsilon_{1}\\ .\\ .\\ \varepsilon_{10} \end{array}\right]$$

where, E(***ε****t*) = 0 and Cov (*f t*,***ε****t*) = 0.

Replacing α and β with θ the above Generalized Method of Moments equation will be transformed into the following quadratic equation.

(3) $$g{\left(\theta \right)}^{T}W_{g}\left(\theta \right),where......g\left(\theta \right)=\left(\frac{1}{T}\right)\sum_{t=1}^{T}Z_{t}\left(\theta \right)$$

The GMM moment's condition s are defined at the true values of α and β as,

$$Z_{t}\left(\theta \right)=\left[\begin{array}{l} \left(R_{tx}-\alpha -\beta_{ft}\right)\\ \left(R_{tx}-\alpha -\beta_{ft}\right)⊗\left[f_{t}\right] \end{array}\right]$$

## 4 Empirical findings and discussion

### 4.1 Descriptive statistics

We begin our analysis by reporting the descriptive statistics of the quintiles constructed according to the firm characteristics namely market value representing size and market to book ratio representing the value/growth. Table 1 shows the characteristics of all the quintiles formed on the stock fundamental characteristics. The data for all the sorting variables for size and value/growth present substantial variation across the quintiles indicating that these characteristics represent a significant criterion for sorting portfolios. The hedge portfolio is constructed by taking a spread between Q1 and Q5. Where Q1 is the portfolio with the lowest value of the sorting criteria and Q5 is the portfolio with the highest value. The hedge portfolio constructed on market capitalization reports a highly significant annualized return of 20.33% (t = 2.26) and 16.41% (t = 1.85) for equally and value-weighted portfolios respectively. Similarly, the hedge portfolio constructed by sorting market to book ratios also reports a significant annualized excess returns of 19.8% (t = 2.89) and 13.7% (t = 1.65) for equally and value-weighted portfolios respectively. Finally, there is no trend found in CAPM beta corresponding to the portfolio returns for any of the firm characteristics which indicate that there is no relation between the risk and the return measured through CAPM beta. It suggests that the mean-variance framework fails to gauge the risk-adjusted returns instating a need to follow other asset pricing models to explain the risk-adjusted portfolio returns.

**Table 1:** Performance Measures of Portfolio Quintiles Constructed on Various characteristics.

	Q1 (low)	Q2	Q3	Q4	Q5 (High)	Q1-Q5	t-statistics
Size (Market Value)							
Average MV	23	296	1599	5783	50400	−50377	−19.57
EW returns (% p.a)	7.85	1.37	1.28	1.47	1.01	6.83	2.26
VW returns (% p.a)	3.60	1.40	1.44	1.43	0.67	2.93	1.85
MV (PKR-thousand)	1.03	13.3	73.1	266.4	2408.6	−2407.6	−22.70
CAPM Beta	0.19	0.2	0.22	0.26	0.2	−0.01	
**Market to Book Ratio**							
Average MTBV	−0.2	0.76	1.28	2.04	7.87	−8.07	−24.28
EW returns (% p.a)	2.54	1.75	1.51	1.28	0.89	1.65	2.89
VW returns (% p.a)	1.84	1.39	0.87	0.61	0.73	1.11	1.65
MV (PKRm)	68.3	191.7	430.6	642.2	1414.5	−1346.1	−37.41
CAPM Beta	0.28	0.21	0.23	0.14	0.26	0.02	

Table 1 shows the descriptive statistics of the style portfolios constructed by using market capitalization and market to book ratios. All non-financial firms listed on PSX have been sorted in ascending order to form quintiles at the end of every month t by taking the previous month firm's characteristics. Q1 in the table reports the portfolio with the lowest equity characteristics and Q5 represents the portfolio with the highest equity characteristics. The no of companies in the quintiles are identical. Q1-Q5 denotes the hedge portfolio that is longing Q1 and shorting Q5 for variables. The excess returns are reported as monthly percentage returns for equally weighted and value-weighted portfolios denoted as EW and VW respectively. MV is the sum of the market values of the shares included in each portfolio (in PKR m). CAPM beta measures the sensitivity of the returns of value-weighted portfolios towards the returns of the market portfolio.

### 4.2 Risk-adjusted asset pricing

After the analysis of descriptive characteristics for all the variables, we evaluate the performance of the style portfolios by first taking into consideration the risk factors associated with the style variables namely size and value. We evaluate the performance of the style portfolios we use Capital Asset pricing model (CAPM), Fama-French three-factor model (FF3) and Fama-French five factors model (FF5).

(4) $$E\left({Rp}_{i}\right)=\alpha_{CAPM}+\beta_{m}\left(R_{m}-R_{f}\right)+\varepsilon_{i}$$

(5) $$E\left(Rp_{i}\right)=\alpha_{FF3}+\beta_{3}\left(R_{m}-R_{f}\right)+\beta_{s}SMB+\beta_{v}HML+\varepsilon_{i}$$

(6) $$E\left({Rp}_{i}\right)=\alpha_{FF5}+\beta_{m}\left(R_{m}-R_{f}\right)+\beta_{s}SMB+\beta_{h}HML+\beta_{R}RMW+\beta_{c}CMA+\varepsilon_{i}$$

where E ($Rp_{i}$) is the expected return on portfolio i, α presents the alpha coefficients given by the CAPM, FF3 and FF5 factor models, SMB is returns on portfolios of small stocks minus big stocks (market capitalization), HML represents high book to market minus low book to market ratio, RMW denotes the robust minus the weak operating profitability factor and CMA represents the difference between the investment returns of firms with conservative and aggressive investment styles.

The testable hypotheses based on the above asset pricing models are that alpha coefficients do not have positive and significant alphas because the asset pricing models explains the variations in the asset returns and thus we formulate our hypotheses as follows:

H1 $\alpha_{CAPM}=0$

H2 $\alpha_{FF3}=0$

H3 $\alpha_{FF5}=0$

Table 2 shows the monthly alpha coefficients of the quintiles constructed by the market capitalization (MC) and the market to book ratios (MTBV). Panel A and panel B reports the results of equally-weighted (EW) and value-weighted (VW) portfolios respectively. The Q1 is reported to have highly significant alpha coefficients in all asset pricing models for both EW and VW specifications. The small-cap portfolio earns an abnormal monthly excess returns from 4.62% to 6.72% in EW and from 2.63% to 2.39% in VW specifications. This indicates the prominent small size effect in the Pakistani market. Q2 also shows significant abnormal excess returns in CAPM and FF3 models in both EW and VW specifications however the significance disappears gradually as we move to large-cap stocks. Similar to the size quintiles, the value quintiles also show significant alphas in the lowest quintiles but for the EW specification only. As we move towards the growth stocks the premiums disappear. The model's intercept is further authenticated by using Wald test to evaluate the significance of asset pricing model explaining the behavior of portfolios constructed on the basis of style variables. The Wald test rejects the null hypothesis of joint coefficients to be zero for all the quintiles constructed by market capitalization in the CAPM and FF3 models while it fails to reject the hypotheses for the market to book value portfolios as indicated by their p values of 0.032, 0.095 and 0.288 respectively under EW specification. The Wald test produces the similar results for the VW specification.

**Table 2:** Asset Pricing of Style portfolios through Asset Pricing Models.

Quintiles constructed by MC						
	Panel A: Equally-Weighted Portfolios			Panel B: Value-Weighted Portfolios		
	CAPM	FF3	FF5	CAPM	FF3	FF5
Q1	4.62%	8.00%	6.72%	2.63%	3.00%	2.39%
	(3.74)***	(2.20)***	(2.05)**	(3.25)***	(2.90)***	(2.20)**
Q2	0.95%*	1.00%	0.92%	0.92%	1.00%	0.97%
	(1.76)	(1.64)*	(1.40)	(1.70)*	(1.69)*	−1.39
Q3	0.94%	1.00%	1.00%	0.98%	1.00%	0.79%
	(1.95)*	(1.52)	(1.27)	(2.02)**	(1.56)	(1.32)
Q4	1.02%	1.00%	0.81%	0.89%	1.00%	0.71%
	(1.99)*	(1.40)	(1.23)	(1.75)*	(1.28)	(1.06)
Q5	0.70%	0.00%	0.28%	0.33%	0.00%	0.03%
	(1.43)	(0.70)	(0.45)	(0.66)	(0.36)	(0.04)
Chi Sq.	12.19	9.36	6.19	11.96	9.32	6.39
Prob.	0.032	0.095	0.288	0.035	0.096	0.269
**Quintiles constructed by MTBV**						
	**Panel A: Equally-Weighted Portfolios**			**Panel B: Value-Weighted Portfolios**		
Q1	2.18%	2.00%	1.91%	1.10%	1.00%	1.14%
	(3.00)***	(2.18)**	(2.03)**	(1.39)	(1.33)	(1.10)
Q2	1.32%	1.00%	0.66%	0.92%	1.00%	0.26%
	(2.37)**	(1.40)	(0.95)	(1.44)	(0.93)	(0.88)
Q3	1.21%	1.00%	0.77%	0.51%	0.00%	−0.33%
	(2.28)**	(1.49)	(1.13)	(0.86)	(0.18)	(0.45)
Q4	0.90%	1.00%	0.76%	0.30%	0.00%	0.06%
	(1.90)*	(1.26)	(1.29)	(0.57)	(0.32)	(0.08)
Q5	0.64%	0.00%	0.59%	0.14%	0.00%	0.36%
	(1.44)	(0.74)	(1.10)	(0.31)	(0.43)	(0.57)
Chi Sq.	8.64	7.12	9.00	4.9	3.66	5.08
Prob.	0.124	0.211	0.109	0.428	0.599	0.406

Table 2 reports the performance of the quintile portfolios constructed as equal and value-weighted portfolios sorted on market capitalization and market to book value for the sample period of 14 years from Jan 2004–Dec 2017. All the non-financial firms listed on PSX since Jan 2003 are sorted at month t in ascending order according to MV and MTBV. Q1 represents the quintile with the lowest value of the fundamental characteristics and Q5 represents the highest value of the firm's characteristics. Q1-Q5 represents the hedge portfolio. The table reports the monthly alphas with the t statistics reported in parenthesis. The chi-square statistic refers to the Wald test evaluating the overall significance of the model; p-values are reported below the statistic.

Next, we regress the excess returns of style portfolios on business cycle regimes constructed by applying Harding and Pagan [52] approximation to the Bry-Boschan (B.B.) algorithm [53] to identify turning points in the Pakistan's Industrial production index. We are interested to know whether the style premiums vary across various regimes as specified by phases of the business cycle. We use the following model to incorporate business cycle fluctutations in our model.

(7) $$E\left({RP}_{i}\right)=\alpha +\beta_{m}\left(R_{m}-R_{f}\right)+\gamma_{b}TP$$

where TP represents the turning points that is peaks and troughs. The periods from the peak to trough is called recession and it is given a dummy value of 1 while the period from trough to peak is an expansionary period and it is given a value of 0. We attempt to determine whether the style premiums vary according to different regimes as specified by phases of the business cycle. Thus we hypothesize that style premiums do not have any impact due to business cycle phases. We formulate this hypothesis as follows:

H4 $\gamma_{b}=0$

Table 3 shows the results of the performance of style portfolios by using a model that incorporates business cycle fluctuations. The lowest quintile constructed by MV values show the highest significant alpha coefficients indicating that the smallest size portfolios earn the greatest abnormal excess returns compared to mid and large cap stocks which are not explained by the business cycle phases. The quintiles constructed by MTBV also show significant alpha values however, the abnormal returns are smaller as compared to the size portfolios. We further observe that the excess returns decrease as we move from small-cap stocks (9.2%, 5.5%) to large-cap stocks (1.7%, 1.5%) and from value (4.1%, 2.9%) to growth stocks (1.4%, 1.5%) in both EW and VW specifications. We also report that the size and value premiums have negative relationship with the recession which indicates that recession depresses the size premium. We also document the higher impact of recession on small firms and value stocks as compared to big firms and growth stocks respectively. The Wald test rejects the null hypothesis of joint coefficients to be zero for the Value weighted portfolio while it fails to reject the hypotheses for the Equal weighted portfolios constructed by the market capitalization as indicated by their p-values of 0.033 and 0.164 respectively. The Wald test also fails to reject the hypotheses for joint coefficients to be zero for the Equal weighted portfolios constructed by market to book value as indicated by the p-values of 0.0025 whereas it rejects the hypotheses for value weighted portfolios as indicated by their p-values of 0.1356.

**Table 3:** Asset pricing of style portfolios through business cycle phases.

Quintiles constructed by MV						
Equally Weighted Portfolio				Value Weighted Portfolio		
	Intercept	RM	Bus_Cycles	Intercept	RM	Bus_Cycles
Q1	0.092	0.743	−0.068	0.055	0.106	−0.053
t-value	(2.76)***	(1.06)	(-2.15)**	(3.27)***	(0.77)	(-3.08)***
Q2	0.022	0.143	−0.028	0.023	0.150	−0.030
t-value	(2.77)***	(1.88)*	(-2.61)***	(3.01)***	(2.06)**	(-2.77)***
Q3	0.023	0.141	−0.032	0.024	0.169	−0.033
t-value	(3.53)***	(2.44)**	(-3.38)***	(3.60)***	(2.89)***	(-3.38)***
Q4	0.023	0.219	−0.031	0.023	0.216	−0.031
t-value	(3.32)***	(3.30)***	(-3.06)***	(3.25)***	(3.20)***	(-3.02)***
Q5	0.017	0.164	−0.026	0.015	0.155	−0.028
t-value	(2.64)***	(2.43)**	(-2.58)***	(2.32)**	(2.23)**	(-2.60)***
Chi Sq. 7.86				Chi Sq. 17.76		
Prob. 0.1640				Prob. 0.0033		
**Quintiles Constructed by MTBV**						
**Equally Weighted Portfolio**				**Value Weighted Portfolio**		
	Intercept	RM	Bus_Cycles	Intercept	RM	Bus_Cycles
Q1	0.041	0.191	−0.049	0.029	0.219	−0.038
t-value	(4.17)***	(2.10)**	(-3.31)***	(2.63)***	(2.10)**	(-2.31)**
Q2	0.026	0.234	−0.032	0.019	0.173	−0.022
t-value	(3.54)***	(2.85)***	(-2.83)***	(2.33)**	(1.60)	(-1.57)
Q3	0.024	0.191	−0.033	0.016	0.189	−0.027
t-value	(3.47)***	(2.57)***	(-3.26)***	(1.94)*	(2.34)**	(-2.29)**
Q4	0.022	0.194	−0.032	0.016	0.092	−0.028
t-value	(3.54)***	(2.86)***	(-3.44)***	(2.33)**	(1.24)	(-2.40)**
Q5	0.014	0.148	−0.019	0.015	0.213	−0.029
t-value	(2.35)**	(2.30)**	(-2.15)**	(2.32)**	(3.06)***	(-2.89)***
Chi Sq. 18.42				Chi Sq. 18.40		
Prob. 0.0025				Prob. 0.1356

Table 3 reports the performance of equal and value-weighted portfolios constructed by market capitalization and market to book value for the sample period of 14 years from Jan 2004–Dec 2017. All the non-financial firms listed on PSX since Jan 2003 are sorted at month t in ascending order according to MV and MTBV values. Q1 represents the quintile with the lowest value of the fundamental characteristics and Q5 represents the highest value of the firm's characteristics. Q1-Q5 represents the hedge portfolio. The table reports the monthly alphas with the t statistics reported in parenthesis. The chi-square statistic refers to the Wald test evaluating the overall significance of the model; p-values are reported below the statistic.

#### 4.2.1 Pricing two way sorted style portfolios

We also test the robustness of style portfolios by applying two way sorting. First, we sort the stocks in descending order (based on their market capitalization) and classify the top 50% as big-cap stocks and bottom 50% stocks as small-cap stocks. Then, we sort the stocks in both groups according to their market to book value ratios in the descending order. The top 33% of the stocks under each big and small-cap groups are classified into growth stocks, middle 33% in the neutral and bottom 33% in the value stocks. Thus, we have come up with (2 × 3) 6 dimensions; small-growth (SG), small-neutral (SN), small-value (SV), big-growth (BG), big-neutral (BN) and big-value (BV). We are not only interested in value or size per se but also in examining how the performance of size portfolios varies among value, neutral and growth stocks. We further make portfolios using the different size and value groups. The SPV portfolio captures the size effect among value portfolios and is calculated as SV-BV. SPN and SPG are also constructed likewise to measure the excess returns variation among neutral and growth stocks. SPN-G, SPV-N and SPV-G can be constructed to measure the return differential of neutral over growth, value over neutral and value over growth among size portfolios. The definitions of all the portfolios constructed using this approach are delineated in Table 4.

**Table 4:** Definitions of premium portfolio constructed on size and value.

Style Portfolios	Construction formula
VP	((SV + BV) - (SG + BG))/2
SP	((SV + SN + SG) - (BV + BN + BG))/3
VPS (VP: small-cap portfolio)	SV – SG
VPB (VP: big-cap portfolio)	BV – BG
SPV (SP: value portfolio)	SV–BV
SPN (SP: neutral portfolio)	SN–BN
SPG (SP: growth portfolio)	SG – BG
SPN-G	SPN – SPG
SPV-N	SPV–SPN
SPV-G	SPV – SPG

Table 4: This table delineates the construction method of various portfolios. VP and SP denote Value and size premiums, respectively. Next, we construct VP among different size portfolios (market capitalization) and SP among different value portfolios (market to book ratios). Thus, VPS and VPB denote VP among small and big-cap stocks respectively, while SPV means SP among value stocks.

Table 5 reports the monthly alpha coefficients of the portfolios constructed as equal and value-weighted portfolios sorted on the size and then subsequently sorted on the value factor for the sample period of 14 years from Jan 2004–Dec 2017. All the non-financial firms listed on PSX since Jan 2003 are sorted at month t in ascending order according to market capitalization and then on market to book values. (***), (**) and (*) denote the significance at the 1%, 5% and 10% respectively.

**Table 5:** Asset pricing of two-way sorted style portfolios through asset pricing models.

Style portfolio	Panel A: Equally-weighted portfolios			Panel B: Value- weighted portfolios		
	CAPM	FF3	FF5	CAPM	FF3	FF5
SV	2.69%***	2.48%***	2.57%***	1.98%**	1.89%**	1.90%*
SN	1.32%*	1.15%*	0.94%**	1.20%**	0.90%	0.62%
SG	1.26%*	1.06%*	1.10%***	1.23%**	1.03%*	1.09%*
BV	1.26%	0.75%	0.36%*	1.00%	0.60%	0.18%
BN	0.97%	0.88%	0.82%*	0.36%	0.17%	−0.09%
BG	0.34%	0.13%	0.37%	0.31%	0.23%	0.12%
SPV	1.44%***	1.73%***	2.21%**	0.98%*	1.28%**	1.72%**
SPN	0.35%	0.27%	0.12%	0.85%**	0.73%	0.71%
SPG	0.93%**	0.93%**	0.73%***	0.92%*	0.80%	0.97%*
SPV-N	1.08%**	1.46%**	2.08%**	0.13%	0.56%	1.01%
SPV-G	−0.57%	−0.66%	−0.61%	0.06%	0.48%	0.75%
SPN-G	0.93%**	0.93%**	0.73%***	−0.07%	−0.07%	−0.26%
SP	0.91%***	0.98%***	1.02%***	0.91%**	0.94%**	1.13%**
VP	1.51%	1.15%	1.10%**	1.03%	0.85%	0.56%
VPS	1.43%**	1.42%**	1.47%**	0.75%	0.86%	0.81%
VPB	0.92%	0.63%	−0.01%**	0.69%	0.37%	0.06%

Panel A and B of Table 5 reports the monthly alphas of CAPM, Fama-French three factors (FF3) and Fama-French five factors (FF5) models for the equally and value weighted portfolios respectively. The size portfolios among value and growth dimensions are highly to moderately significant in the EW and VW specifications in all the asset pricing models while the SN portfolio is insignificant in the FF3 and FF5 factor models. We also document the significant size effect in the value and growth hedge portfolios (SPV and SPG) however the neutral stocks don't fare any better among size portfolios. The portfolios that go long in value and short in neutral and the portfolios that go long in neutral and short in growth stocks are significant in the EW specification only. We further observe that the size premium (SP) is highly significant in both the equal and value weighted portfolios. The value premium is also prominent in the small-cap equally weighted portfolios. The significant monthly alphas indicate that the asset pricing models fail to explain the abnormal excess returns and thus the portfolios with significant alphas suggest that size and value premium are the contributory factors in constructing profitable portfolios. We observe that the monthly CAPM alpha coefficients decrease as we move from the value (2.69%, 1.98%) to growth stocks (1.26%, 1.23%) among small-cap stocks under both EW and VW specifications. The FF3 and FF5 coefficients also follow the same pattern. Likewise, the monthly alpha coefficients decline when we move from SPV to SPG portfolios inferring that the size premium is most prominent in the value stocks followed by growth. The neutral stocks however do not earn any significant returns. We further notice that the portfolios constructed by high values of market capitalization or market to book ratios report the lower premiums as compared to low market-cap and market to book values. Reporting the value premium across different market-caps we find that it exists only in the small-cap stocks.

We further include the business cycle phases in our analysis to investigate if the style premiums are the result of some endogenous risk factors. Table 6 provides the strong evidence of style portfolio premiums after incorporating the business cycle phases. Further, the negative coefficients on business cycle phases suggest the negative premiums in the recession. We thus conclude that the style portfolios earn abnormal returns which are the results of the fundamental characteristics the portfolios possess and are not fully explained by the business cycle movements.

**Table 6:** Asset Pricing of two way sorted Style portfolios through business cycles.

	Equally-weighted			Value- weighted		
	Intercept	RM	Bus_Cyc	Intercept	RM	Bus_Cyc
SV	0.049	0.194	−0.053	0.041	0.137	−0.051
	(4.71)***	(2.05)**	(-3.24)***	(3.92)***	(1.40)	(-3.08)***
SN	0.028	0.160	−0.036	0.030	0.182	−0.042
	(4.30)***	(2.31)**	(-3.35)***	(4.23)***	(2.73)***	(-3.83)***
SG	0.025	0.217	−0.030	0.025	0.234	−0.031
	(3.98)***	(2.94)***	(-3.16)***	(3.71)***	(2.87)***	(-3.14)***
BV	0.030	0.193	−0.043	0.026	0.178	−0.038
	(3.78)***	(2.08)**	(-3.05)***	(3.16)***	(1.84)*	(-2.62)***
BN	0.022	0.171	−0.030	0.016	0.155	−0.030
	(3.41)***	(2.39)**	(-2.59)***	(2.35)**	(2.16)**	(-2.49)**
BG	0.013	0.151	−0.023	0.013	0.148	−0.023
	(2.31)**	(2.12)**	(-2.42)**	(2.01)**	(2.02)**	(-2.05)**
SPV	0.019	0.001	−0.010	0.015	−0.041	−0.013
	(2.08)**	(0.01)	(-0.87)	(1.88)*	(-0.69)	(-1.23)
SPN	0.006	−0.011	−0.006	0.014	0.027	−0.012
	(1.32)	(-0.20)	(-0.94)	(2.63)***	(0.54)	(-1.39)
SPG	0.013	0.066	−0.008	0.013	0.086	−0.008
	(2.74)***	(1.74)*	(-1.29)	(1.78)*	(0.97)	(-0.78)
SPV-N	0.012	0.012	−0.003	0.002	−0.067	−0.001
	(1.50)	(0.19)	(-0.32)	(0.19)	(-1.00)	(-0.05)
SPV-G	−0.006	−0.077	0.001	0.003	−0.126	−0.005
	(-1.11)	(-1.24)	(0.16)	(0.28)	(-1.37)	(-0.35)
SPN-G	0.013	0.066	−0.008	0.001	−0.059	−0.004
	(2.74)***	(1.74)	(-1.29)	(0.14)	(-0.68)	(-0.37)
SP	0.012	0.019	−0.008	0.014	0.024	−0.011
	(2.73)***	(0.45)*	(-1.35)	(2.78)***	(0.50)	(-1.52)
VP	0.033	0.160	−0.044	0.027	0.115	−0.040
	(4.16)***	(1.89)	(-3.02)***	(3.09)***	(1.06)	(-2.51)**
VPS	0.024	−0.023	−0.022	0.016	−0.097	−0.020
	(2.69)***	(-0.27)	(-1.76)*	(1.84)*	(-1.09)	(-1.59)
VPB	0.018	0.043	−0.020	0.013	0.029	−0.015
	(3.17)***	(0.60)	(-2.16)**	(2.09)**	(0.50)	(-1.65)*

Table 6 reports the performance of equal and value-weighted portfolios sorted on the size and then subsequently sorted on the value factor for the sample period of 14 years from Jan 2004–Dec 2017. All the non-financial firms listed on PSX since Jan 2003 are sorted at month t in ascending order according to market capitalization and then on market to book values. (***), (**) and (*) denote the significance at the 1%, 5% and 10% respectively.

## 5 Conclusion

This study provides evidence that equity-style investing can be a profitable strategy for portfolio investment in the Pakistani equity market. The research employs a methodology that incorporates firm-specific characteristics and macroeconomic variables to assess the performance of style portfolios over time. The study finds that style premiums exist in the Pakistani equity market and that abnormal returns for style portfolios are consistently earned, even after accounting for the impact of macroeconomic factors. Small firms tend to outperform big firms and hence taking a long position for the quintiles with the smallest market value and taking a short position on the quintile with the biggest market value stocks would help investors earn higher returns. Similarly, value firms tend to outperform growth stocks and hence taking a long position for the quintile with the smallest MTBV earns the excess returns above the market. We also document the significant size effect in the two-way sorted portfolios in all asset pricing models. Our results are robust after incorporating business cycle phases. The results of the study have several implications for investors and academics alike. For investors, the findings suggest that style investing can be a viable strategy for generating higher returns, especially during economic expansions. The methodology employed in the research provides a framework for constructing style portfolios based on firm-specific characteristics and assessing their performance over time. For academics, the study adds to the literature on efficient market hypothesis by exploring the profitability of equity style investing in the context of the Pakistani equity market. The methodology of two way sorted style portfolios used in the research enhances the robustness of the analysis and provides a framework for future studies. In summary, this study contributes to the understanding of equity style investing in emerging markets by providing insights into the profitability of style portfolios in the Pakistani equity market. The findings suggest that investors can benefit from incorporating style investing into their portfolio strategy, and that further research is needed to better understand the underlying causes of style premiums.

The current research adds to the existing literature on equity style investing and its profitability in the Pakistan Stock Exchange, which has not been extensively studied before. The findings demonstrate that style portfolios consistently generate abnormal returns, indicating that style investing can be profitable in the Pakistani market. The study's use of a two-way sorted style portfolio methodology and multiple factors to evaluate the size and value premiums enhances the robustness of the analysis and provides a framework for future research that examines the impact of business cycles on style premiums. The research provides insights into the persistence of size and value premiums across varying business conditions, which could benefit investors and policymakers. Moreover, the study highlights the limitations of the Fama-French three and five factor models, as well as the business cycles model, in explaining the abnormal returns of style portfolios. This contributes to the ongoing debate about market efficiency and the validity of asset pricing models.

Furthermore, the study also has some limitations. For example, the research only examines a limited number of equity styles based on size and value/growth, which may not reflect the full range of potential equity styles that could produce different results. Moreover, the study does not consider the impact of transaction costs or other investment expenses, which can affect the overall profitability of style investing. Additionally, the research does not provide a clear explanation for the underlying factors that contribute to the profitability of style investing in the Pakistani market, leaving room for further investigation and interpretation.

## Author contribution statement

Muhammad Kashif: Conceived and designed the experiments; Performed the experiments.

Sumaira Chamadia: Performed the experiments; Contributed reagents, materials, analysis tools or data.

Farhan Ahmed; Juan E. Trinidad Segovia: Analyzed and interpreted the data; Wrote the paper.

## Data availability statement

Data will be made available on request.

## Declaration of competing interest

The authors declare that they have no known competing financial interests or personal relationships that could have appeared to influence the work reported in this paper.

## Figures and Tables

**Table A-1:** Summary Statistics of Sorting Variables

Quantiles	Mean	Standard Deviation	Skewness	Kurtosis
Panel A: Market Capitalization				
Q1	23	36	2.94	9.15
Q2	296	429	2.31	5.71
Q3	1599	1846	1.67	1.89
Q4	5783	5994	1.60	1.55
Q5	50400	33483	0.79	−0.85
Panel B: Market to Book values				
Q1	−0.20	0.51	0.16	−1.28
Q2	0.76	0.30	0.15	−1.18
Q3	1.28	0.42	0.00	−1.32
Q4	2.04	0.60	−0.10	−1.33
Q5	7.87	2.48	0.11	−1.10

**Table A-2:** Correlation Matrix of Explanatory Variables

Variables	Rmrf	SMB	HML	RMW
SMB	−0.293			
HML	−0.199	0.266		
RMW	−0.385	−0.088	0.277	
CMA	0.119	0.323	0.170	−0.308

## Appendix – A

Table A-1 reports the summary statistics of Sorting Variables; Market capitalization (Panel A) and Market to Book values (Panel B). Q1 in the table reports the portfolio with the lowest equity characteristics and Q5 represents the portfolio with the highest equity characteristics.

Table A-2 reports the correlation matrix of the explanatory variables. Rmrf denotes the excess returns on market portfolio, SMB is returns on portfolios of small stocks minus big stocks (market capitalization), HML represents high book to market minus low book to market ratio, RMW denotes the robust minus the weak operating profitability factor and CMA represents the difference between the investment returns of firms with conservative and aggressive investment styles.

## Appendix – B

### Assumptions of GMM Model

The Generalized Method of Moments (GMM) model relies on a set of assumptions [54] to ensure that the estimators obtained through the method are consistent and efficient. Some of the key assumptions of the GMM model include:

1. Moment conditions: The model is based on a set of moment conditions, which are functions of the observed data and the unknown parameters. These moment conditions are assumed to be well-defined, have finite variance, and satisfy a set of regularity conditions.
2. Exogeneity: The moment conditions are assumed to be orthogonal to the error term, which means that they are uncorrelated with the underlying unobserved error term.
3. Moment restrictions: The moment conditions are assumed to be sufficient to identify the unknown parameters of the model. In other words, the number of moment conditions should be greater than or equal to the number of unknown parameters.
4. Validity: The moment conditions should be valid, meaning that they should be based on valid economic theory or empirical evidence.
5. Large sample size: The GMM estimator relies on asymptotic theory, which assumes that the sample size is large enough to ensure that the estimator is consistent and efficient.
6. Stationarity: The model assumes that the underlying process generating the data is stationary, meaning that the statistical properties of the data do not change over time.

## References

[1] Chan H., Docherty P. (2016). Momentum in Australian style portfolios: risk or inefficiency?. Account. Finance.

[2] Chou P.-H., Ko K.-C., Yang N.-T. (Jan. 2019). Asset growth, style investing, and momentum. J. Bank. Finance.

[3] Fama E.F., French K.R. (1992). The cross-section of expected stock returns. J. Finance.

[4] Lakonishok J., Shleifer A., Vishny R.W. (1994). Contrarian investment, extrapolation, and risk. J. Finance.

[5] Lewellen J. (Jan. 2002). Momentum and autocorrelation in stock returns. Rev. Financ. Stud..

[6] Chen N.-F., Roll R., Ross S.A. (1986). Economic forces and the stock market. J. Bus..

[7] Fama E.F., French K.R. (1989). Business conditions and expected returns on stocks and bonds. J. Financ. Econ..

[8] Daniel K., Titman S. (1997). Evidence on the characteristics of cross sectional variation in stock returns. J. Finance.

[9] King B.F. (1966). Market and industry factors in stock price behavior. J. Bus..

[10] Farrell J.L. (1974). Analyzing covariation of returns to determine homogeneous stock groupings. J. Bus..

[11] Rong W. (2013). http://etheses.dur.ac.uk/7324/.

[12] Brinson G.P., Singer B.D., Beebower G.L. (May 1991). Determinants of portfolio performance II: an update. Financ. Anal. J..

[13] Hansen J. (1992). US equity investment styles. Secur. Anal. J..

[14] Sharpe W.F. (Jan. 1992). Asset allocation: management style and performance measurement. J. Portfolio Manag..

[15] Lintner J. (1965). Security prices, risk, and maximal gains from diversification. J. Finance.

[16] Sharpe W.F. (1964). Capital asset prices: a theory of market equilibrium under conditions of risk. J. Finance.

[17] Jensen-Gaard C. (2014).

[18] Basu S. (1977). Investment performance of common stocks in relation to their price-earnings ratios: a test of the efficient market hypothesis. J. Finance.

[19] Banz R.W. (Mar. 1981). The relationship between return and market value of common stocks. J. Financ. Econ..

[20] Acharya V.V., Pedersen L.H. (Aug. 2005). Asset pricing with liquidity risk. J. Financ. Econ..

[21] Amihud Y. (Jan. 2002). Illiquidity and stock returns: cross-section and time-series effects. J. Financ. Mark..

[22] Carlson M., Fisher A., Giammarino R. (2004). Corporate investment and asset price dynamics: implications for the cross-section of returns. J. Finance.

[23] Garleanu N., Panageas S., Yu J. (2012). Technological growth and asset pricing. J. Finance.

[24] Asness C., Frazzini A., Israel R., Moskowitz T.J., Pedersen L.H. (Sep. 2018). Size matters, if you control your junk. J. Financ. Econ..

[25] An C., Cheh J.J., Kim I. (2017). Do value stocks outperform growth stocks in the U.S. Stock market?. J. Appl. Finance Bank.

[26] Clark E., Qiao Z. (May 2020). The value premium puzzle, behavior versus risk: new evidence from China. Q. Rev. Econ. Finance.

[27] Chan K.C., Chen N.-F. (1991). Structural and return characteristics of small and large firms. J. Finance.

[28] Rosenberg B., Reid K., Lanstein R. (1985). Persuasive evidence of market inefficiency. J. Portfolio Manag..

[29] Fama E.F., French K.R. (Feb. 1993). Common risk factors in the returns on stocks and bonds. J. Financ. Econ..

[30] Chan K., Hameed A., Tong W. (Jun. 2000). Profitability of momentum stragegies in the international equity markets. J. Financ. Quant. Anal..

[31] Dichev I.D. (1998). Is the risk of bankruptcy a systematic risk?. J. Finance.

[32] Dimson E., Marsh P. (1999). Murphy's law and market anomalies. J. Portfolio Manag..

[33] M. Michou, S. Mouselli, and A. W. Stark, Fundamental Analysis and the Modelling of Normal Returns in the UK, Social Science Research Network, Rochester, NY, SSRN Scholarly Paper ID 1607759, May 2010. Accessed: March. 2, 2020. [Online]. Available: https://papers.ssrn.com/abstract=1607759.

[34] Barry C.B., Goldreyer E., Lockwood L., Rodriguez M. (2002). Robustness of size and value effects in emerging equity markets, 1985–2000. Emerg. Mark. Rev..

[35] Fama E.F., French K.R. (Sep. 2012). Size, value, and momentum in international stock returns. J. Financ. Econ..

[36] Horowitz J.L., Loughran T., Savin N.E. (Aug. 2000). Three analyses of the firm size premium. J. Empir. Finance.

[37] Oertmann P. (2000).

[38] Ahn D.-H., Min B.-K., Yoon B. (May 2019). Why has the size effect disappeared?. J. Bank. Finance.

[39] Zhang L. (2005). The value premium. J. Finance.

[40] Zhang Q.J., Hopkins P., Satchell S., Schwob R. (Dec. 2009). The link between macro-economic factors and style returns. J. Asset Manag..

[41] Hou K., van Dijk M.A. (Jul. 2019). Resurrecting the size effect: firm size, profitability shocks, and expected stock returns. Rev. Financ. Stud..

[42] Bolten S.E., Weigand R.A. (1998). The generation of stock market cycles. Financ. Rev..

[43] DeStefano M. (2004). Stock returns and the business cycle. Financ. Rev..

[44] Kwag S.-W., Whi S. (2006). Value investing and the business cycle. J. Financ. Plann..

[45] Dahlquist M., Harvey C.R. (2001). https://papers.ssrn.com/abstract=795376.

[46] Bernanke B.S., Gertler M. (2015).

[47] Hahn J., Lee H. (Jun. 2006). Yield spreads as alternative risk factors for size and book-to-market. J. Financ. Quant. Anal..

[48] Kiyotaki N., Moore J. (Apr. 1997). Credit cycles. J. Polit. Econ..

[49] Campbell J.Y. (1996). Understanding risk and return. J. Polit. Econ..

[50] Perez-Quiros G., Timmermann A. (2000). Firm size and cyclical variations in stock returns. J. Finance.

[51] Fama E.F., French K.R. (1996). Multifactor explanations of asset pricing anomalies. J. Finance.

[52] Harding D., Pagan A. (2002). Dissecting the cycle: a methodological investigation. J. Monetary Econ..

[53] Bry G., Boschan C. (1971). https://ideas.repec.org//b/nbr/nberbk/bry_71-1.html.

[54] Hansen L.P. (1982). Large sample properties of generalized method of moments estimators. Econometrica.

